# Supplementary material for: Classification and functional characterization of regulators of intracellular STING trafficking identified by genome-wide optical pooled screening
Source: bioRxiv. 2024 Apr 9:2024.04.07.588166. Preprint. [Version 1] doi: 10.1101/2024.04.07.588166 (PMC11030420; doi:10.1101/2024.04.07.588166)
Supplement: Supplement 10 [file NIHPP2024.04.07.588166v1-supplement-10.pdf]

bioRxiv preprint doi: https://doi.org/10.1101/2024.04.07.588166; this version posted April 9, 2024. The copyright holder for this preprint (which was not certified by peer review) is the author/funder, who has granted bioRxiv a license to display the preprint in perpetuity. It is made available under aCC-BY 4.0 International license.

Supplementary Information

Table S1. Genome-wide optical pooled screen mean per-gene features.
Table S2. Genome-wide OPS SVM classifier results.
Table S3. Meta-analysis by information content results.
Table S4. Dimensionality reduction clusters from genome-wide screen.
Table S5. Secondary screen mean per-gene features for HeLa cells.
Table S6. Secondary screen mean per-gene features for BJ1 cells.
Table S7. Secondary screen dimensionality reduction results and unstimulated vs 4 hour PHATE potential distances.
Table S8. List of sgRNAs used in the study
Table S9. List of antibodies used in the study
Supplementary Figures 1-5.

bioRxiv preprint doi: https://doi.org/10.1101/2024.04.07.588166; this version posted April 9, 2024. The copyright holder for this preprint (which was not certified by peer review) is the author/funder, who has granted bioRxiv a license to display the preprint in perpetuity. It is made available under aCC-BY 4.0 International license.

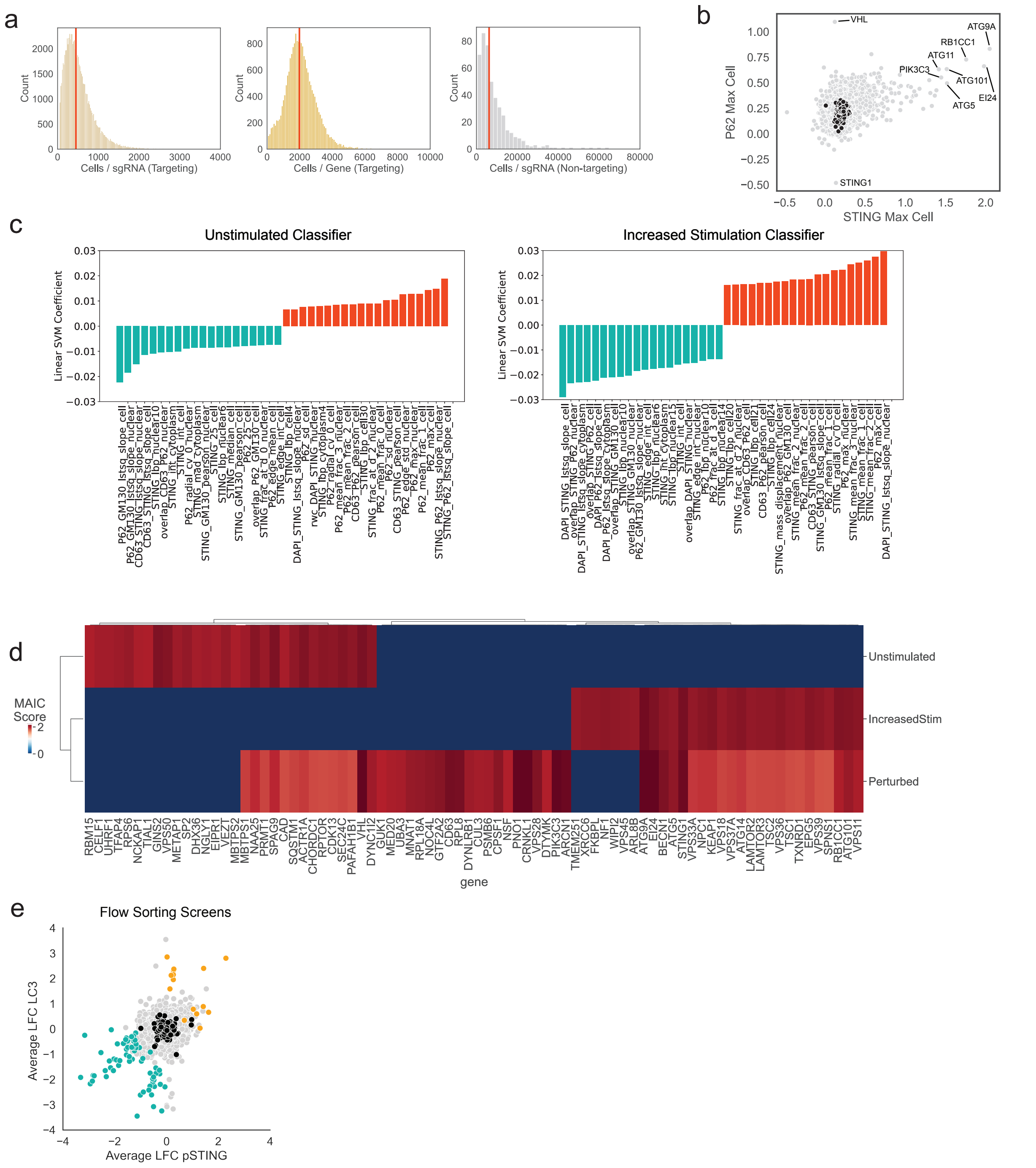

Figure S1. (A) Distribution of number of cells in the screen for each sgRNA or gene for targeting sgRNAs and non-targeting controls. Medians highlighted in red. **(B)** Scatterplot of STING per-cell maximum intensity and p62 maximum intensity for each gene in the screen. Black dots indicate non-targeting control sgRNAs. **(C)** Top and bottom 20 feature weights for SVM unstimulated and increased stimulation classifiers. **(D)** MAIC scores for top 30 genes (by overall MAIC score) for each OPS SVM classifier. **(E)** Correlation of log2 fold change (LFC) for pSTING and LC3 in STING flow cytometry screens. Orange: genes that increased both LC3 and pSTING at p < .001 in both screens; blue: genes that decreased both metrics at the same significance.

bioRxiv preprint doi: https://doi.org/10.1101/2024.04.07.588166; this version posted April 9, 2024. The copyright holder for this preprint (which was not certified by peer review) is the author/funder, who has granted bioRxiv a license to display the preprint in perpetuity. It is made available under aCC-BY 4.0 International license.

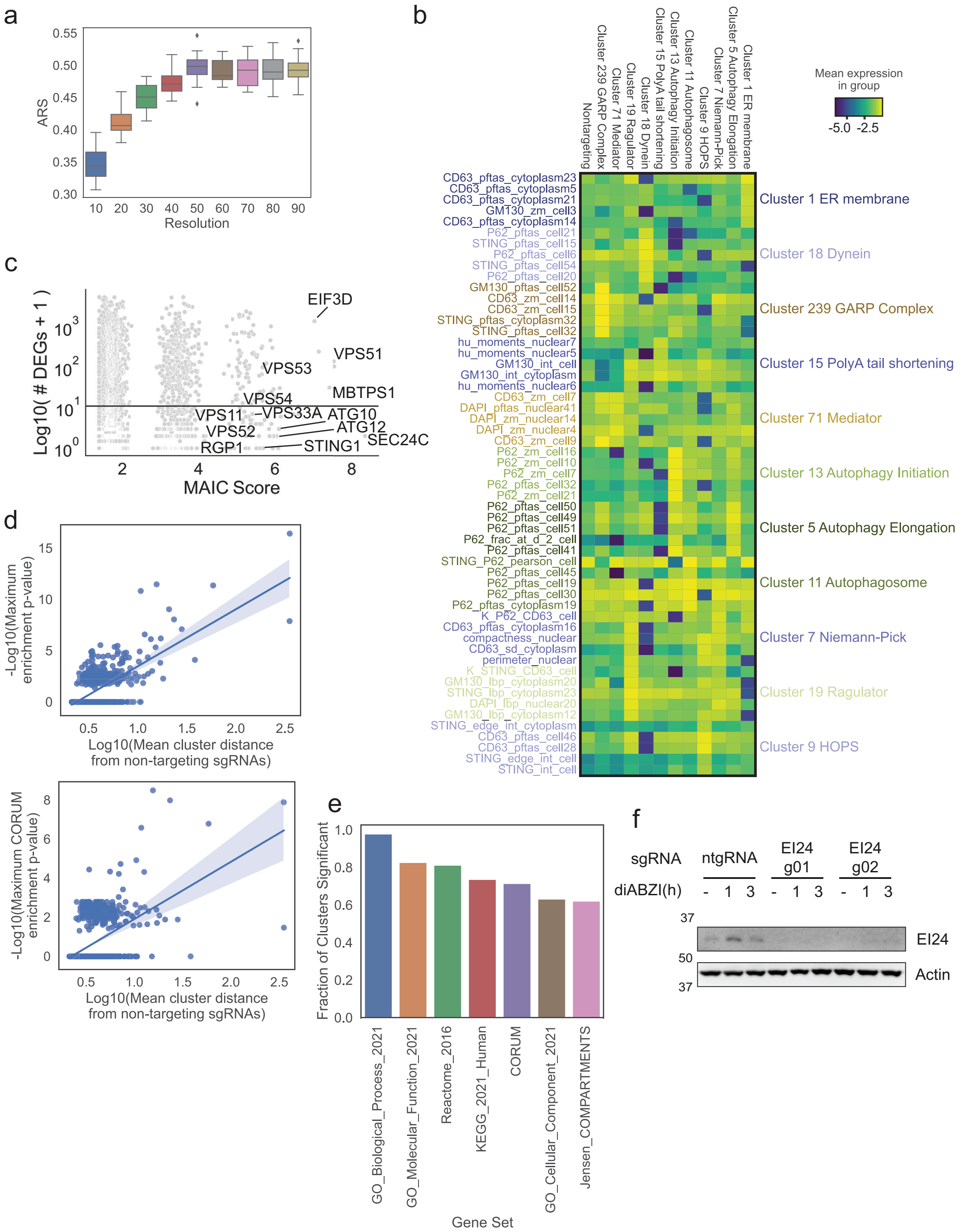

Figure S2. (A) Adjusted Rand score for Leiden clustering at different resolutions. **(B)** Top 5 features significantly differentiating clusters highlighted in Figure 2. **(C)** MAIC Score plotted against number of DEGs for genes included in the genome-scale Perturb-seq K562 dataset. **(D)** Scatterplots of mean cluster distance from non-targeting sgRNAs (PHATE potential distance) against the maximum enrichment p-value from Enrichr (GO, Reactome, KEGG, Jensen COMPARTMENTS and CORUM datasets, top) or from CORUM alone (bottom) **(E)** Fraction of clusters (among clusters with >1 gene and >20% of genes not non-targeting sgRNAs) that had at least one significant term as calculated by Enrichr for the noted categories. **(F)** Immunoblot of the indicated proteins in BJ1 fibroblasts transduced with a control guide (ntgRNA) or with EI24 sgRNAs and stimulated with 1μM diABZI for the indicated times.

bioRxiv preprint doi: https://doi.org/10.1101/2024.04.07.588166; this version posted April 9, 2024. The copyright holder for this preprint (which was not certified by peer review) is the author/funder, who has granted bioRxiv a license to display the preprint in perpetuity. It is made available under aCC-BY 4.0 International license.

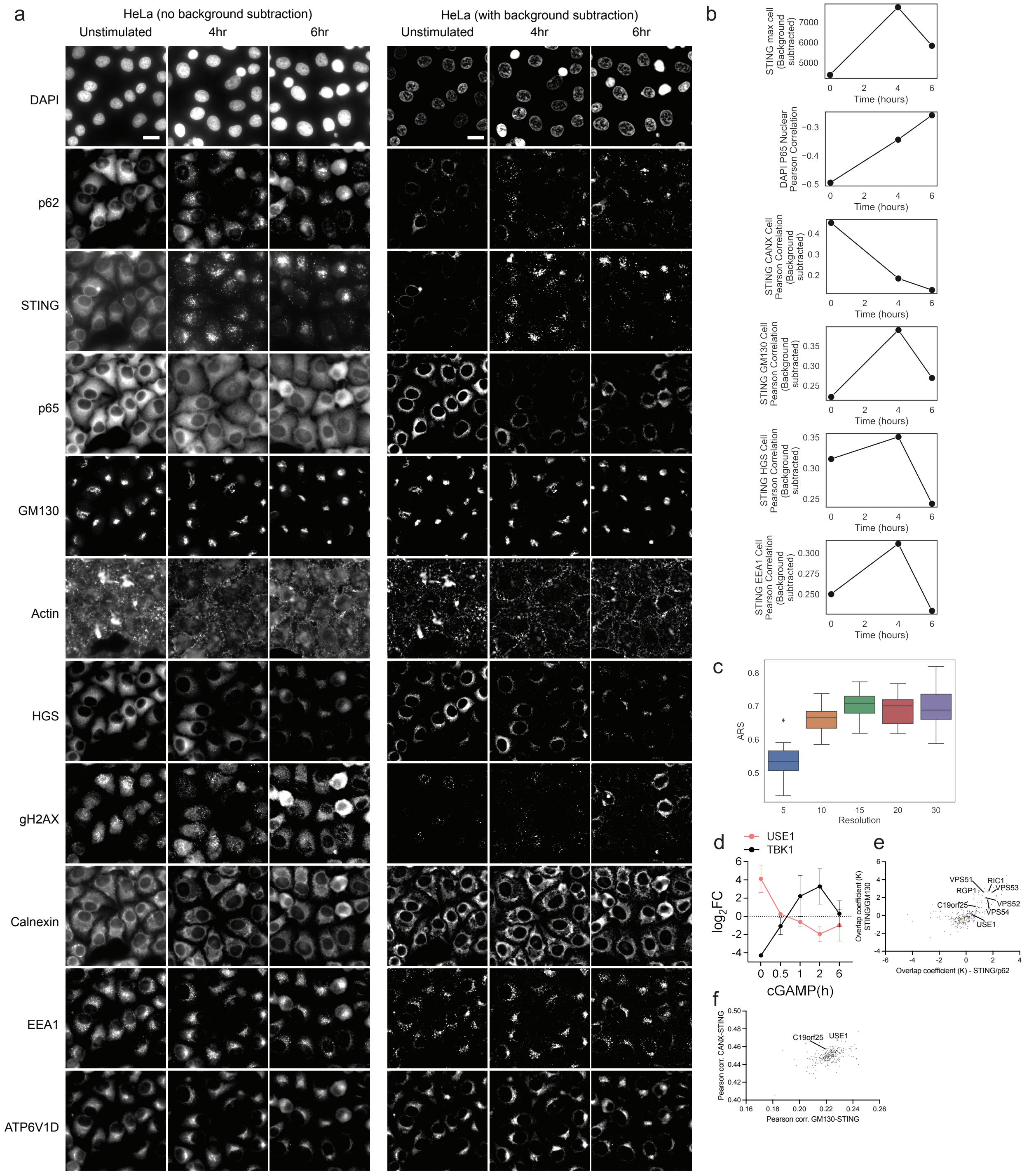

Figure S3. (A) Selected fields of view from secondary screens for HeLa cells, all channels shown. Scale bar 20 µm. **(B)** HeLa secondary screen non-targeting mean feature values across time. **(C)** Adjusted Rand score (ARS) for Leiden clustering at different resolutions. **(D)** log2 fold change (log2FC) enrichment of the indicated proteins in the STING-TurboID datasets at the indicated timepoints post cGAMP stimulation. **(E)** Overlap coefficient for the indicated channels in integrated deviation from ntgRNA from secondary OPS. **(F)** Pearson correlation of the indicated channels in unstimulated cells from the secondary OPS.

bioRxiv preprint doi: https://doi.org/10.1101/2024.04.07.588166; this version posted April 9, 2024. The copyright holder for this preprint (which was not certified by peer review) is the author/funder, who has granted bioRxiv a license to display the preprint in perpetuity. It is made available under aCC-BY 4.0 International license.

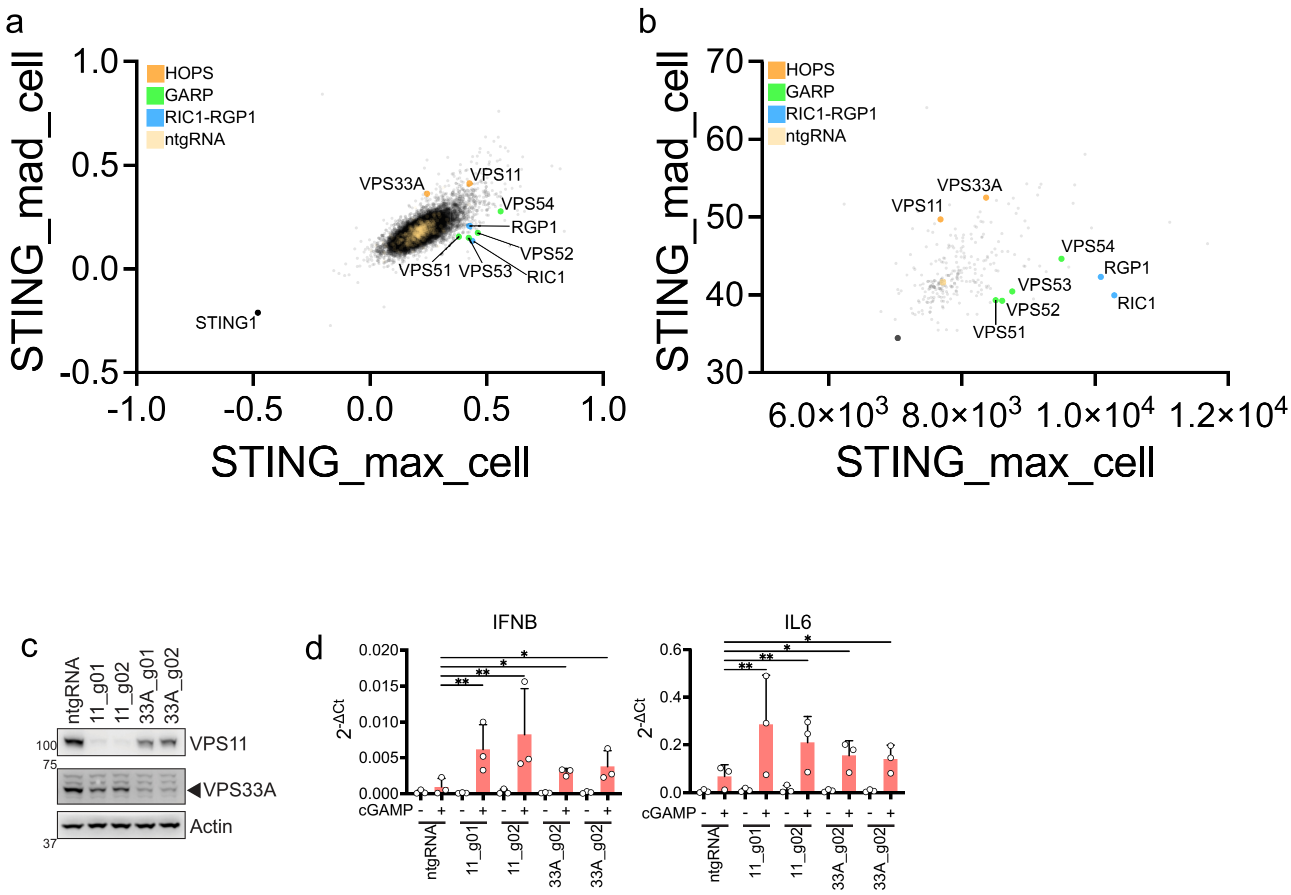

Figure S4. (A) STING_max and STING_mad extracted features correlation in the genome-wide OPS in HeLa cells. Specific subunits of complexes of interest are indicated in color. STING1 is highlighted in black. Non-targeting control sgRNAs are indicated in yellow. Pixel intensity is normalized to ntgRNAs. **(B)** STING_max and STING_mad extracted features correlation in the genome-wide OPS in HeLa cells. Specific subunits of complexes of interest are indicated in color. STING1 is highlighted in black. Non-targeting control sgRNAs are indicated in yellow. Background subtracted pixel intensity values are plotted. **(C)** Immunoblot of the indicated proteins in 293T STING-mNG transduced with control (ntgRNA) or VPS11 or VPS33A sgRNAs. One blot representative of n=3 blots. **(D)** Raw $2^{-\Delta Ct}$ values related to Fig. 4e.

bioRxiv preprint doi: https://doi.org/10.1101/2024.04.07.588166; this version posted April 9, 2024. The copyright holder for this preprint (which was not certified by peer review) is the author/funder, who has granted bioRxiv a license to display the preprint in perpetuity. It is made available under aCC-BY 4.0 International license.

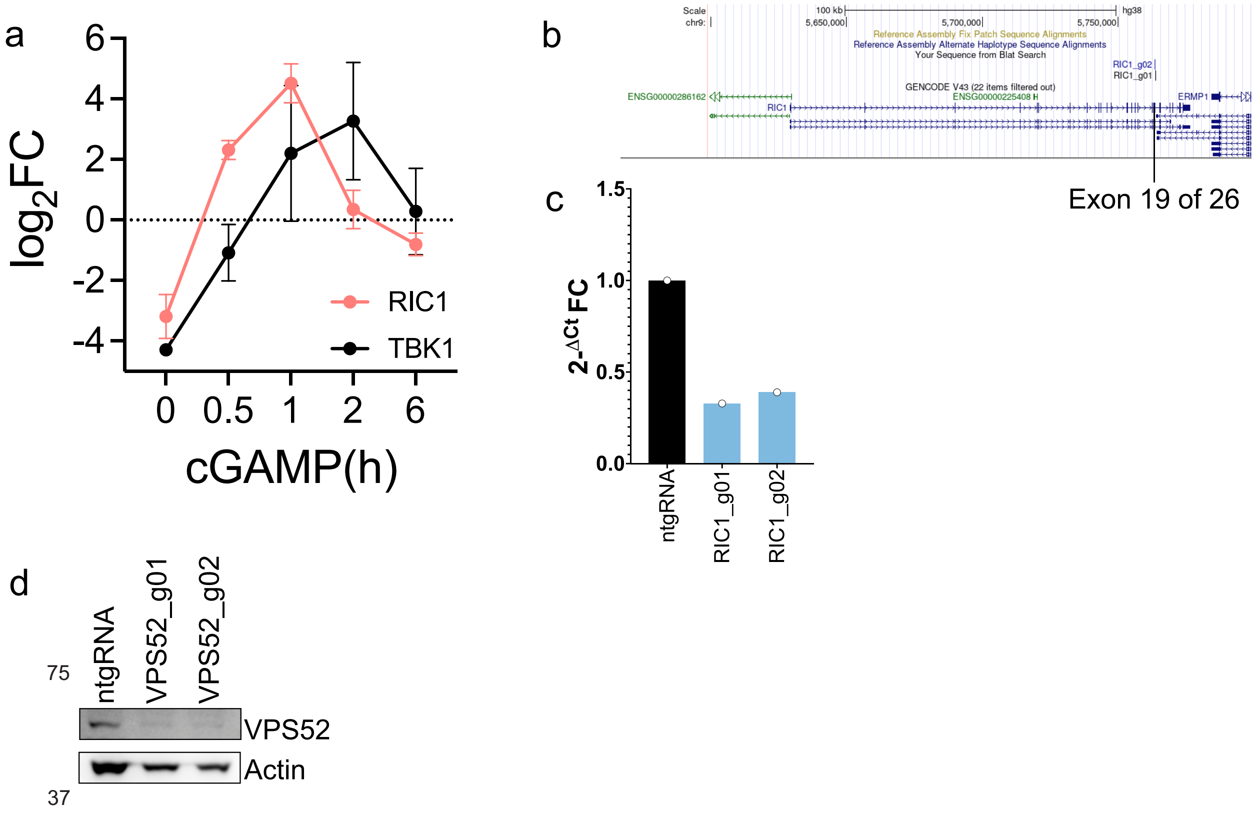

Figure S5. (A) log2 fold change (log2FC) enrichment of the indicated proteins in the STING-TurboID datasets at the indicated timepoints post cGAMP stimulation. **(B)** Location of the two RIC1 targeting sgRNAs used in this paper visualized in UCSC Genome browser. While we could not identify a reliable antibody for RIC1, both guides targeted the gene in exon 19 of 26 at >50-55nt from the exon-exon junction potentially triggering Non-Sense Mediated Decay[72]. **(C)** qPCR of RIC1 expression in 293T STING-mNG transduced with a control (ntgRNA) or RIC1 targeting guides as in b). **(D)** Immunoblot of the indicated proteins in 293T STING-mNG transduced with a control (ntgRNA) or VPS52 targeting sgRNAs.
